# High-Resolution Ultrasound for Complications of Botulinum Toxin Use: A Case Series and Literature Review

**DOI:** 10.7759/cureus.63232

**Published:** 2024-06-26

**Authors:** Claudia Gonzalez, Jaime Rengifo, Paola Macias-Arias, Valeria Duque-Clavijo, Brian D Noreña-Rengifo

**Affiliations:** 1 Radiology, Rosario University, Bogota, COL; 2 Dermatology, Pontifical Bolivarian University, Medellín, COL; 3 Dermatology, Federico Lleras Acosta ESE University Hospital Dermatological Center, Bogota, COL; 4 Dermatology, Universidad de los Andes, Bogota, COL; 5 Radiology, Universidad de Antioquia, Medellín, COL

**Keywords:** nodules, facial asymmetry, ptosis, hematoma, pseudoaneurysm, botulinum toxin, high-resolution ultrasound

## Abstract

Botulinum toxin (BTX) has revolutionized both aesthetic and therapeutic medicine by selectively inhibiting acetylcholine release at the neuromuscular junction, inducing localized muscle relaxation. However, its use can be associated with various complications. As a diagnostic modality, high-resolution ultrasound can better characterize these complications.

Here, we present four clinical cases of complications associated with the application of BTX, along with their corresponding ultrasonographic findings. In this study, cases were selected randomly, irrespective of the timing of BTX injections, to illustrate a spectrum of complications observed in clinical practice.

Despite its benefits, BTX can have adverse effects ranging from mild to severe, including aesthetic and functional complications, such as hematoma, ptosis, facial asymmetry, nodules, or pseudoaneurysm. High-resolution ultrasound emerges as a crucial tool in the multidisciplinary management of these complications, allowing for accurate evaluation and effective therapeutic guidance.

## Introduction

Botulinum toxin (BTX) has emerged as a revolutionary tool in the fields of aesthetic medicine and therapeutics. Its fundamental role in a wide spectrum of clinical applications comes from its ability to selectively inhibit the release of acetylcholine at the neuromuscular junction, inducing localized muscle paralysis. From correcting facial wrinkles to treating debilitating neurological disorders, BTX has been proven to be a therapeutically and aesthetically relevant option. In aesthetic medicine, despite being a very safe procedure, its use is not exempt from the appearance of various complications. Currently, with the positioning of high-resolution ultrasound as the diagnostic modality par excellence for characterizing the various complications in aesthetic medicine, it is possible to understand more precisely the behavior of complications derived from the use of BTX and contribute, with diagnostic images, relevant information for multidisciplinary management [1-3]. We present four clinical cases with complications secondary to the use of BTX, along with clinical-ultrasound correlation. In this study, the inclusion of cases was not consecutive. Patients were selected randomly, irrespective of the timing of BTX injections, to illustrate a spectrum of complications observed in clinical practice. The reported cases aim to highlight various potential adverse outcomes associated with the use of BTX.

## Case presentation

Case 1

**Figure 1:**
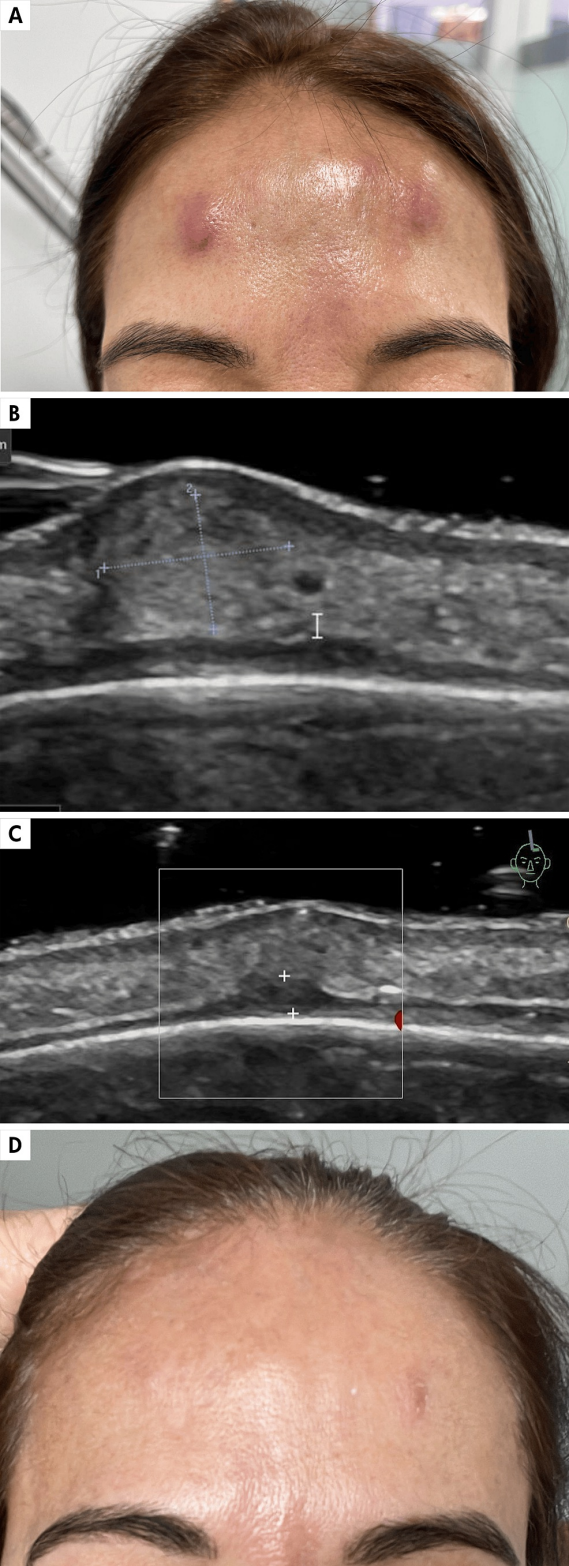
Inflammatory nodules seen with high-resolution gray-scale ultrasound. A. Inflammatory nodules in the frontal region after botulinum toxin injection. B. High-resolution gray-scale ultrasound image, axial view, showing an inflammatory nodule in the dermal-subcutaneous layer, which is hyperechoic with partially defined borders (delimited by calipers +), causing displacement of the epidermis superiorly. C. High-resolution gray-scale ultrasound image, axial view, immediately below the inflammatory nodule (n), there is a thickening of the frontalis muscle with irregular borders suggestive of myositis (delimited by calipers +). D. Clinical image of the frontal region after completing treatment showing near-complete resolution of the nodules.

A 61-year-old female presented on December 12, 2023, with a prior history of BTX injection. She reported the onset of painful erythematous nodules in the injection sites, manifesting 10 days post-procedure. Despite several prescribed antibiotic regimens, none were effectively completed until the final course. Initially, treatment comprised a seven-day course of amoxicillin-clavulanic acid BID, yielding only partial improvement. Subsequently, antibiotic therapy was modified to cefadroxil 500 mg BID, but because of inadequate response, it was changed to ciprofloxacin 500 mg BID for 12 days. However, upon cessation of medication, a recurrence of the nodules ensued, accompanied by an increase in size and local erythema. Seeking a second opinion, diagnostic considerations encompassed inflammatory/infectious nodules post-injection versus foreign body granulomatous reaction (Figure 1A). High-resolution ultrasound imaging (Figures 1B, 1C) favored the diagnosis of infectious myositis and dermal-subcutaneous inflammatory nodules consistent with lobular panniculitis. Treatment entailed moxifloxacin 400 mg BID for 14 days, supplemented with an oral corticosteroid pulse regimen, starting with prednisolone 0.5 mg/kg/day and gradually tapering. Following completion of therapy, the patient exhibited significant clinical amelioration, with complete resolution of the lesions (Figure 1D).

Case 2

**Figure 2:**
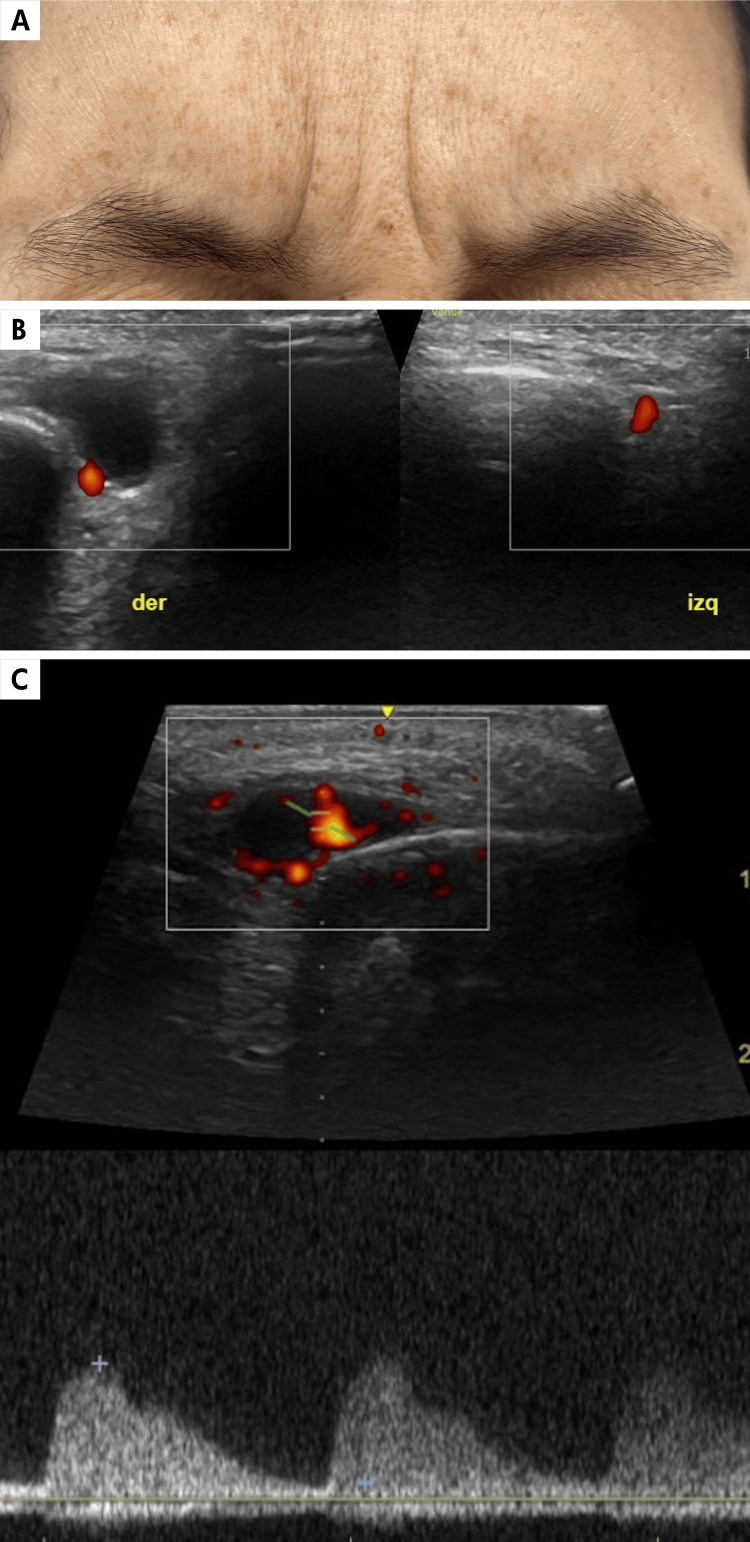
Pseudoaneurysm seen with high-resolution gray-scale and Doppler color duplex ultrasound. A. Clinical image of a patient with a sensation of right supraorbital mass becoming more evident with facial contraction. B. Comparative axial gray-scale and Doppler color duplex ultrasound image of the supraorbital region. The right and left supraorbital arteries are normal. On the right side, above the artery, there is an anechoic, oval-shaped, pseudo-cystic image (delimited by calipers +). C. Spectral waveform confirming arterial flow within the lesion. Corresponds to a pseudoaneurysm.

A 45-year-old female patient presented on March 7, 2023, with a sensation of a mass in the right supraorbital region immediately after a BTX injection. Clinically interpreted as hematoma, the lesion persisted 10 months post-BTX application, was mildly painful, and increased in size with Valsalva maneuvers (Figure 2A). Doppler ultrasound demonstrates an oval-shaped image with an arterial flow inside, corresponding to a pseudoaneurysm (Figures 2B, 2C).

Case 3

**Figure 3:**
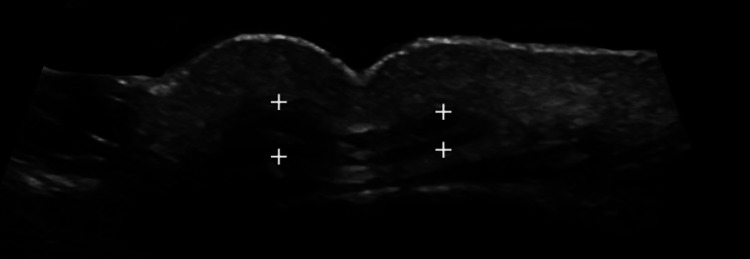
Asymmetry seen with high-resolution gray-scale ultrasound. High-resolution ultrasound, axial grayscale image, demonstrating the procerus muscle (delimited by calipers +) with asymmetry in its thickness. During dynamic exploration with contraction on the right side, the fibers show increased thickness, explaining the marked wrinkle toward the right of the midline and the undesired cosmetic component.

A female patient presented on October 25, 2023, having experienced an undesirable cosmetic result after the application of BTX in the glabellar region. Persistent wrinkling and asymmetry appeared immediately after application, which worsened over time. At four months, a high-resolution ultrasound was performed, revealing an asymmetry in the thickness of the procerus muscle, and explaining the undesirable cosmetic outcome. Figure 3 depicts the ultrasonographic changes.

Case 4

**Figure 4:**
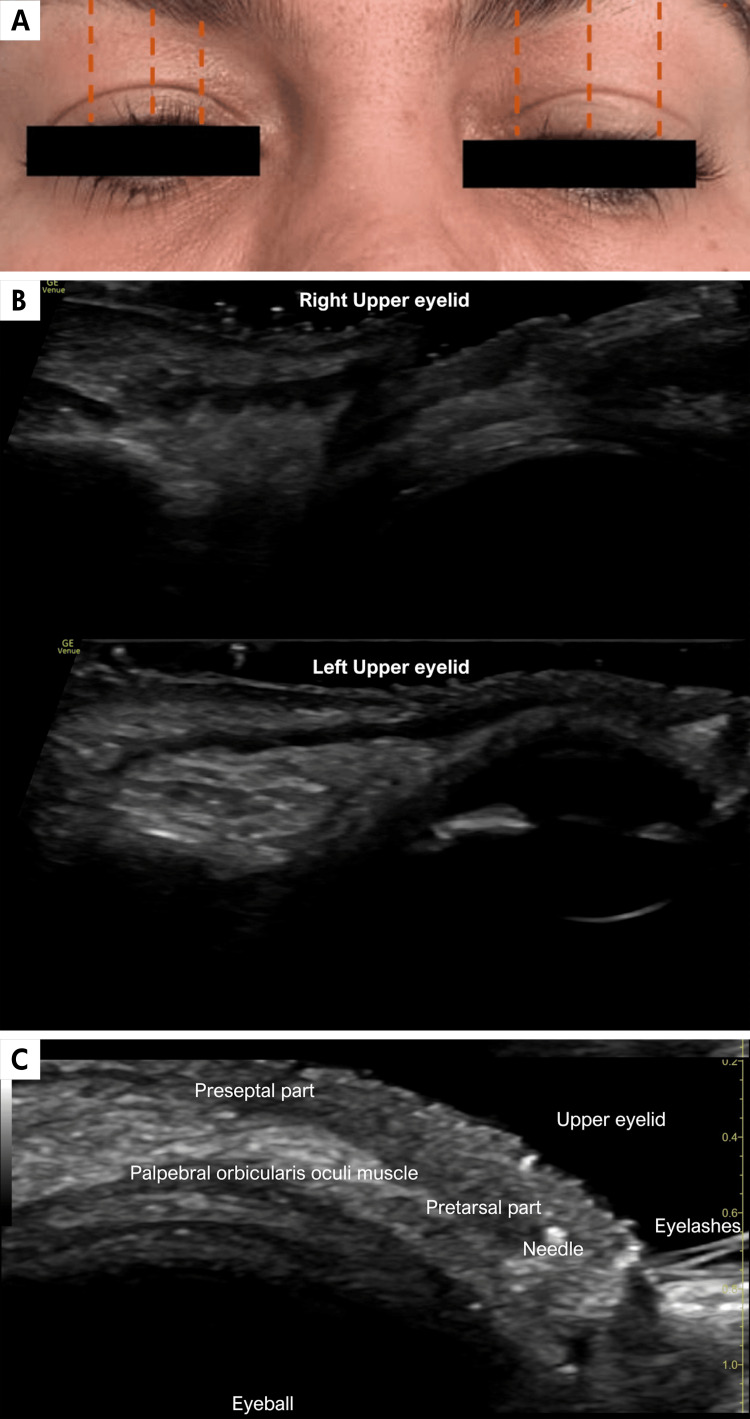
Ptosis seen with high-resolution gray-scale ultrasound. A. Clinical image showing asymmetry in the length of the orange lines, demonstrating left ptosis. B. High-resolution ultrasound with a linear probe (18 MHz) revealing incomplete contraction of the orbicularis oculi muscle in the left eyelid secondary to an incorrect technique in the botulinum toxin injection on the left side. Normal and complete contraction is observed on the right side. C. Ultrasound-guided injection of botulinum toxin into the pretarsal part of the orbicularis oculi muscle in the left upper eyelid for treatment.

A young female patient, who received BTX injections in the glabella and forehead seven days prior, presented on December 1, 2023, with ptosis of the left upper eyelid (Figure 4A). High-resolution ultrasound revealed an incomplete contraction of the pretarsal portion of the orbicularis muscle, explaining the observed ptosis (Figure 4B). As part of the treatment, it was decided to inject one unit of BTX into the external preseptal portion of the upper eyelid, achieved with total precision under ultrasound guidance (Figure 4C).

## Discussion

BTX was initially introduced into clinical practice by Dr. Alan B. Scott in the 1970s to treat strabismus, with Food and Drug Administration (FDA) approval granted in 1989. Its applications broadened significantly after Jean and Alastair Carruthers discovered its effects on facial wrinkles in 1987, which led to FDA approval for cosmetic uses in 1992. Today, BTX is the most widely used cosmetic procedure in the United States, appreciated for its versatility and safety across a range of clinical and aesthetic applications [4].

Research has led to the development of six FDA-approved BTX formulations, predominantly featuring BTX-A because of its long-lasting effects and straightforward production process [5]. Advances in biotechnology, such as next-generation sequencing and recombinant DNA technology, have further expanded potential applications, identifying new variants and enhancing their therapeutic efficacy [4].

Recently, ultrasound technology has acquired prominence in enhancing BTX applications. It has become important in the guided administration of injectables, including the use of advanced techniques for BTX and the management of potential complications. A systematic review highlighted its utility in pre-injection evaluations, guiding injections, and post-injection monitoring. This technique facilitates the visualization of muscles, tendons, and other internal structures, significantly improving the safety and efficacy of injections. It provides noninvasive and immediate diagnostic capabilities for cosmetic procedures [6,7].

Mechanism of action

Produced by the bacterium *Clostridium botulinum*, BTX disrupts acetylcholine release at neuromuscular junctions, effectively causing temporary muscle paralysis. The heavy chain of the toxin binds to the cholinergic neuron membrane, facilitating cellular internalization through endocytosis. The light chain then acts to inhibit acetylcholine release, with effects peaking at day 14 post-injection. While nerve function recovers over time through synaptic regeneration, the paralysis typically lasts between three and four months, though this can vary based on multiple factors, including the specific treatment protocol [8].

Aesthetic uses

In aesthetic medicine, BTX is approved for treating forehead lines, glabellar lines, lateral canthal lines, and severe axillary hyperhidrosis. Off-label uses have grown considerably, contributing to its popularity in procedures such as brow lifting, nasal bridge line reduction, and nasal tip lifting. It is also employed in the lower face to address gummy smiles and vertical perioral wrinkles by relaxing specific facial muscles. Additionally, BTX has been successfully used to improve the appearance of necklines and alter facial contours by reducing masseter muscle size [9]. In the treatment of hypertrophic scars and keloids, BTX has shown benefits in lowering scar size and improving associated symptoms such as itching and pain [10].

Complications

Despite its benefits, it is important to recognize the potential risks associated with BTX use. There are two types of BTX-related adverse effects, which can be transient and benign or potentially serious. Additionally, adverse effects of BTX type A injections versus placebo in the glabellar and frontal regions have been compared and determined to be usually safe. Most of the described complications are mild and transient, with a total complication rate of 16% [11].

The milder and more frequent complications include pain, bruising, or hematoma at the injection site, and are usually transient. Likewise, temporary headaches, paresthesia, and flu-like symptoms are possible. Even mild allergic reactions have been reported, presenting as erythema and edema [12,13].

Regarding cutaneous infections, they are mostly localized at the application site and often present as papules, nodules, plaques, or granulomas [14]. In these less frequent complications, known as inflammatory nodules, as in our first case, high-resolution ultrasound helps confirm aspects such as the depth level of the nodule, involvement of cutaneous structures such as the epidermis, dermis, subcutaneous tissue, and even extension to the muscular layer, as in this case which helped suggest post-application infectious myositis. Likewise, these findings guide toward its possible infectious, inflammatory, granulomatous reaction to a foreign body, or sarcoid-like etiology.

Cases of myositis associated with an inflammatory process caused by BTX injections have been reported in therapeutic settings. Myositis can also result from infections, with only a few cases documented in the literature. In these cases, it could be considered that inflammatory dermal nodules of infectious etiology might cause extension by contiguity to the muscular plane, leading to infectious myositis [14]. Likewise, it should be noted that BTX injections can act as a foreign body, resulting in a foreign-body granulomatous reaction [15]. Pyomyositis is identified on ultrasound as a decrease in muscle echogenicity with an increase in thickness and thickening of the fascia with loss of its margins and increased convexity [15]. On color Doppler or power Doppler, there may be hyperemia in the absence of necrosis or abscesses [16].

On the other hand, when a mass, swelling, or persistent hematoma is documented, ultrasound helps clarify the diagnosis, as observed in case 2, where valuable information was obtained about alterations in the blood vessel wall seen as a pseudoaneurysmal dilation, as the cause of the findings described in this patient. This type of complication has also been described in patients who have undergone midface thread-lifts [17].

Pseudoaneurysms should always be assessed with gray-scale ultrasound, color Doppler, and power Doppler. Gray-scale ultrasound reveals a cystic image adjacent to the supplying artery, which sometimes may have layers or echogenic content because of the presence of hematomas. The diagnosis is established on Doppler ultrasound by the presence of typical swirling internal blood flow, known as the “yin-yang sign,” with the flow in the neck or channel connecting the feeding artery with the pseudoaneurysm, characterized by the presence of systolic and diastolic components known as “to and fro” [18].

At this point, we wish to emphasize that pseudoaneurysms, although rare, can be induced by accidental injections of BTX into the wall of the blood vessel. Several reports have been described in the literature following facial thread-lift, primarily when working in the middle third of the face [17]. Therefore, special caution is recommended in the anatomical areas with greater vascularization [12,19].

Lastly, adverse aesthetic and functional effects can occur because of inadequate muscle response or incorrect placement of BTX. These include ptosis, as highlighted by Case 4, which can occur when treating the frontal muscles for forehead expression lines, as well as the procerus and corrugator muscles for expression lines in the interciliary region. Ptosis can affect both eyelids and eyebrows, with an average of 13.4% of reported cases of brow ptosis [13]. Ectropion, diplopia, xerophthalmia, and lagophthalmos after administration of high doses of BTX in the lateral canthal area are also described. In the case of our patient with ptosis, a dynamic ultrasound examination demonstrated incomplete contraction of the pretarsal portion of the orbicularis muscle, which clarified the diagnosis. Additionally, ultrasound enabled successful treatment as a precise unit of the toxin was placed under ultrasound guidance in the pretarsal portion of the left eyelid edge. The muscle is normally hypoechoic; however, repetitive injections with BTX can lead to decreased thickness and increased echogenicity of the muscle. This has been reported in the masseter muscle, where these findings have been described as fibrosis secondary to BTX application [20].

On the other hand, asymmetry in the lips or their corners is rare but possible following incorrect application that asymmetrically affects portions of the depressor anguli oris muscle or the depressor muscle of the lower lip, with the consequent functional difficulty in speech and eating [13]. The ultrasound-guided treatment of this complication with BTX injection has shown to be more effective and prevents greater diffusion into adjacent muscles, so it should always be considered when ultrasound is available [6].

Serious adverse effects of BTX are uncommon but should not be overlooked. The incidence of these effects is observed to be 33 times higher when BTX is used for therapeutic purposes compared to cosmetic purposes because of higher doses. Among the highlighted complications are dysphagia, dysphonia, and neck weakness, which can arise from deeper application or excessive doses of the toxin in the cervical area, affecting swallowing, phonation, and neck flexion. Severe dysphagia may require temporary dietary changes and may even require enteral nutrition. In severe cases, generalized muscle weakness may develop, compromising the muscles necessary for breathing and leading to respiratory failure. 

Therefore, caution is advised, as this complication may also be a risk associated with other aesthetic procedures. Lastly, cases of BTX-induced anaphylaxis, a potentially life-threatening condition, have been recorded. Systemic dissemination of BTX can occur, necessitating urgent medical attention [12].

## Conclusions

BTX has proven to be an invaluable tool in aesthetic and therapeutic medicine, offering effective solutions for various medical and aesthetic conditions. Although its benefits are clear, it is crucial to recognize and manage the potential associated risks, ensuring that its use is safe and effective. In the event of rare complications or those that do not resolve promptly, a high-resolution ultrasound with Doppler is proposed as the ideal diagnostic tool for proper assessment and timely management.
